# The importance of small natural features in forests—How the overgrowth of forest gaps affects indigenous flower supply and flower‐visiting insects and seed sets of six *Campanula* species

**DOI:** 10.1002/ece3.7965

**Published:** 2021-08-01

**Authors:** Ralf Braun‐Reichert, Sven Rubanschi, Peter Poschlod

**Affiliations:** ^1^ Environmental Station Haus am Strom Untergriesbach Germany; ^2^ Department of Ecology and Conservation Biology Institute of Plant Sciences University of Regensburg Regensburg Germany; ^3^ Terrestrial Ecology Research Group School of Life Sciences Technical University Munich Freising Germany

**Keywords:** bees, *Campanula*, flies, flower visitors, forest management, gap dynamics, pollination

## Abstract

1. The abandonment of historical land‐use forms within forests, such as grazing or coppicing, and atmospheric nitrogen deposition, has led to an increasing overgrowth of forest gaps and canopy closure in forest ecosystems of Central Europe. From 1945 to 2015, 81% of the forest gaps greater than 150 m^2^ within the study area transitioned into a closed forest.
2. This study investigated how the overgrowth process affects flower supply, flower visitors, and reproduction of *Campanula* species. Six native *Campanula* species with different light requirements were used as phytometers.
3. The forest gaps in the studied area are a feature of the historical European cultural landscape. We compared large gaps caused by human activities, small gaps caused by habitat conditions, and closed forests. In eight blocked replicates, each with the three habitat categories, we recorded the flower cover and number of indigenous flowering species in the immediate surroundings, and, of six *Campanula* species, flower visitors and seed production.
4. Forest gaps and their size positively affected the number of flowering plant species in the surrounding area, the number of all flower visitor groups, and the number of seeds produced by all six *Campanula* species. Flower cover in the surrounding area was higher in large gaps, but there was no difference between small gaps and closed forests. Among flower visitors, small bees varied the most between the three habitat categories, and flies varied the least. The effect on the number of seeds produced was particularly strong for three light‐demanding *Campanula* species.
5. The overgrowth of forest gaps negatively affected flower supply, flower‐visiting insects, and seed sets of six *Campanula* species. Forest gaps should be managed to maintain the reproduction of open forest plants and their pollinators.

## Graphical abstract

Nitrogen deposition and abandonment of extensive forms of land use lead to an overgrowth of forest gaps. This study compared the flower supply, flower visitors, and seed set of six *Campanula* species of large and small forest gaps and closed forest. The overgrowth of forest gaps negatively affected flower supply, flower‐visiting insects, and seed sets of six *Campanula* species. (The photograph in the center shows Hoplitis mitis visiting *Campanula persicifolia* and seeds of *C. persicifolia*.)

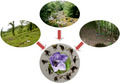

## 1 INTRODUCTION

The pollination process is critical for natural and agricultural systems (Guntern et al., 2014; IPBES, 2016; Klein et al., 2006; Naturkapital Deutschland TEEB, 2018). However, this process is threatened in many parts of the world (Leonhardt et al., 2013; Potts et al., 2010; Sanchez‐Bayo & Wyckhuys, 2019). In Central Europe, changes in land use are considered the most important cause of the decline in pollinators (MEA, 2005; Zurbuchen & Müller, 2012). Multiple studies in Central Europe (Carrié et al., 2017; Hopfenmüller et al., 2014; Tscharntke et al., 2005; Zurbuchen et al., 2010) assessed open landscapes with regard to management intensity, landscape composition, and configuration or distances between nesting and foraging sites, as well as changes in agricultural landscapes. However, changes in forest landscapes and the associated impacts on pollinating insects have received less research attention, even though forests cover 35% of the area of Europe (MCPFE, 2020).

As light is a prerequisite for many plants to flower, many forest plants flower in spring before the trees unfold their leaves. Additionally, forest edges and open forests are richer in flowers (Burgess et al., 2006; Killkenny & Galloway, 2008). Among pollinating insects, bees are among the most important groups (La Salle & Gauld, 1993). However, forests are generally considered sparsely populated by bees (Westrich, 2018; Winfree et al., 2007; Zurbuchen & Müller, 2012). Nevertheless, gaps in forests offer good habitats even for xero‐ and thermophilic bee species due to the abundance of food plants and nesting sites (Fuhrmann, 2007; Kohl & Rutschmann, 2018; Westrich, 2018; Wiesbauer, 2017). As well members of Diptera, in particular, hoverflies, are important flower visitors and pollinators in central Europe (Jauker et al., 2012). According to Ssymank, Doczkal, et al. (2011), 70%–80% of all German hoverfly species are concentrated in open areas or at the edge of forests.

Unprecedented human‐induced changes in the nitrogen (N) cycle in Western Europe in the last century resulted in enormous N deposition from the atmosphere into forest ecosystems (BMEL, 2018; Sutton et al., 2011). Furthermore, the increased atmospheric CO_2_ content promoted optimal use of the available N, thereby increasing tree growth rates over the last 50 years (Ciais et al., 2008; Laubhann et al., 2009; Thomas et al., 2010). The analysis of 23 studies investigating the effect of atmospheric N deposition on plant communities in understory forests showed densification of the canopy and a shift toward shade‐tolerant plant species (Verheyen et al., 2012). In addition to anthropogenic N deposition, the abandonment of traditional land‐use practices has caused a significant improvement in the forest nutrient supply, especially within thin soil layers. Historical land‐use practices, such as forest litter removal, forest pasture, intensive coppicing, or dead wood collection, led to nutrient impoverishment and, thus, low tree growth rates. Open forests with gaps resulted (Gatter, 2004; Poschlod, 2017; Rubner, 1967). Today, approximately 73% of European forests' net annual wood increment is utilized by fellings (MCPFE, 2020). For example, in Germany, forest gaps account for only 2% of the forested area (Hampicke, 2018; Schmalfuß & Aldinger, 2012). In a study of the development of forest gaps in a deciduous forest, our research group (Braun‐Reichert & Poschlod, 2018) revealed an 81% decrease in forest gaps of more than 150 m^2^ from 1945 to 2015 in the study area. Specifically, historical meadows or pastures are no longer used, and clear cuttings and many small sites naturally treeless due to drought and nitrate deficiency are now overgrown because of recent N deposition.

Nevertheless, little is known about the consequences of the overgrowth of forest gaps on the reproduction of entomophilous plants. A suitable approach to experimentally assess habitat quality or the effects of landscape changes on pollinators is to use attractive potted food plants as phytometers (Steffan‐Dewenter et al., 2002; Woodcock et al., 2014; Zurbuchen et al., 2010). Members of the genus *Campanula* are attractive to flower visitors and suitable as phytometer plants due to the quantity and volume of pollen grains and the amount of nectar they produce (Müller et al., 2006). Furthermore, the genus *Campanula* attracts the highest number of oligolectic bee species in Central Europe (Zurbuchen & Müller, 2012). Additionally, flies, especially hoverflies, frequently visit this genus (Hansen & Totland, 2006; Janzon, 1983).

Therefore, this study asked the following questions:

- Is the flower supply as food resources for pollinating insects poorer in a closed forest than in small and large gaps?
- Is the number of *Campanula* flower‐visiting insects lower in a closed forest than in small and large gaps?
- How does the overgrowth of forest gaps affect the seed set of six *Campanula* species with different light demands?

## 2 METHODS

### 2.1 Study area

The study area was “Jochenstein,” the easternmost section of the nature reserve “Donauleiten from Passau to Jochenstein.” The nature reserve is located in the Danube valley in southeast Germany, where it borders Austria. The Danube River cuts into the paragneiss rock within the reserve at a depth of approximately 300 m, and the average slope is 30° (LDBV, 2012).

On the south‐facing slopes, there is a mosaic of different forest communities. In 36% of the study area, forests with *Fagus sylvatica* (Luzulo‐Fagetum) grow on mesophilic sites, and in 30%, forests with *Carpinus betulus* and *Quercus petraea* (Galio‐Carpinetum) grow on dry and warm sites. According to aerial photographs of ADBV (Office for Digitisation, Broadband and Surveying Vilshofen, 2013, unpublished data), forest gaps constituted three per cent of the study area. Openness and gaps of the forests may result from the dry site conditions, exposure to the south in combination with thin layers of soil, scree slopes, and exposed rock outcrops. Identified species include *Origanum vulgare*, *Teucrium scorodonia,* and *Hylotelephium maximum* (Geranio‐Trifolietum with a transition to Sedo‐Scleranthetea). Historical use, such as cuttings, coppicing, removing forest litter, establishing forest pastures, and harvesting leaf fodder, led to further depletion of soil nutrients. Between 2013 and 2015, the study area received 17–19 kg of additional nitrogen deposition per hectare and year (Schaap et al., 2018). Based on extensive surveys of the nearby power plant and the BIOKLIM‐monitoring project (Bässler et al., 2015; Donaukraftwerk Jochenstein, 2012), the insect fauna of the area is known to be particularly species‐rich and thermophilic.

### 2.2 Study design

**FIGURE 1:**
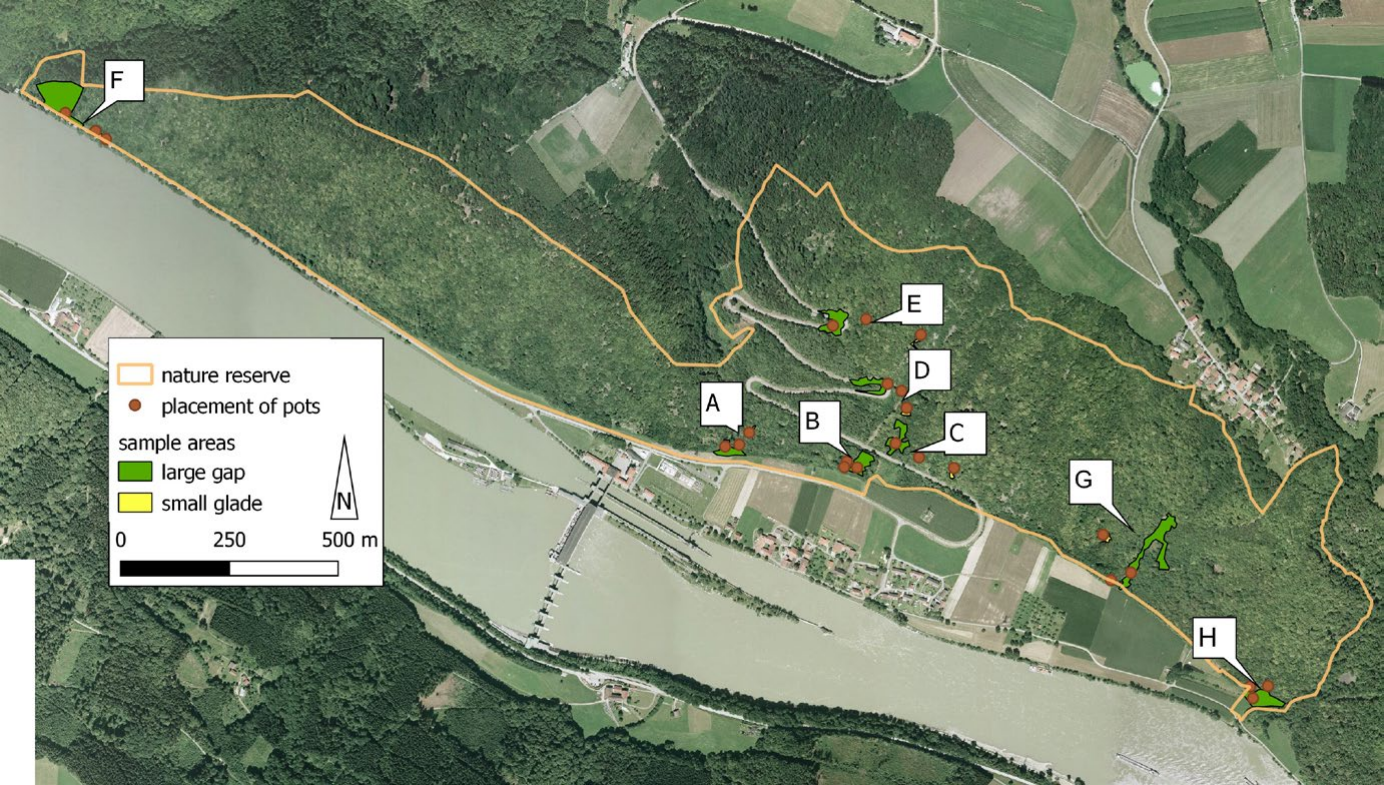
Aerial picture (ADBV, Office for Digitisation, Broadband and Surveying Vilshofen, 2013, unpublished data) of the study area at the Danube river. The beige line delimits the easternmost part “Jochenstein” of the nature reserve “Donauleiten from Passau to Jochenstein.” The red spots show the locations of the pots with the *Campanula* plants. The yellow areas show the small gaps mostly covered by the spots of the pot sites. The green areas show the large gaps. The letters show the short names of the eight blocks A–H

We defined three habitat categories: large gaps (clearings), small gaps (glades), and closed forest. The forest gaps were classified based on their size, measured using aerial photographs (ADBV, Office for Digitisation, Broadband and Surveying Vilshofen, 2013, unpublished data) and QGIS software (QGIS.org, 2018). Small gaps ranged between 32 m^2^ and 375 m^2^ and large gaps between 1,344 m^2^ and 6,008 m^2^ (Table 1). Large gaps were cleared by humans, they resulted for example from road embankments, recent cuttings, nature conservation management measures, or use as meadows (Table 1). Small gaps represent the character of open forest in the study area caused by habitat conditions, namely exposure to the south in combination with thin soil layers, scree slopes, and exposed rock outcrops. We selected sites containing each of these three habitat categories with a maximum distance apart of 210 m to maintain continuity of environmental factors (He et al., 2012). Each site with these three habitat categories formed a statistical block and was replicated eight times (Figure 1). Five blocks were studied in 2015 (blocks A–E) and three blocks in 2016 (blocks F–H; for dates, see Table 2).

**TABLE 1:** Description of the eight study sites by area of the three habitats (in meter^2^) and reasons for the openness of the large gaps

Shortname block	Extension of open area (m^2^)			Reason for openess of large gap
	Closed forest	Small gap	Large gap	
A	0	80	1,344	Meadow
B	0	102	1,664	Clearcutting
C	0	260	2,011	Clearcutting
D	0	317	1,838	Road embankment in bend
E	0	269	2,352	Road embankment in bend
F	0	175	6,008	Clearcutting
G	0	375	4,651	Conservation management measure
H	0	32	2,451	Meadow

**TABLE 2:** The usual dates of the inspection passes for flower resources of the surrounding area and flower‐visiting insects in the three habitats of the respective blocks

blo	param	hab	Pass 1	Pass 2	Pass 3	Pass 4	Pass 5	Pass 6	Pass 7	Pass 8
A	fr & fv	all	13.05.15	31.05.15	11.06.15	25.06.15	05.07.15	15.07.15	24.07.15	03.08.15
B	fr & fv	all	13.05.15	01.06.15	11.06.15	25.06.15	05.07.15	15.07.15	24.07.15	03.08.15
C	fr	lg	13.05.15	01.06.15	14.06.15	25.06.15	05.07.15	15.07.15	24.07.15	03.08.15
C	fr	sg	13.05.15	01.06.15	13.06.15	25.06.15	05.07.15	15.07.15	24.07.15	03.08.15
C	fr	cf	13.05.15	01.06.15	13.06.15	25.06.15	05.07.15	15.07.15	24.07.15	03.08.15
C	fv	all	13.05.15	01.06.15	16.06.15	25.06.15	05.07.15	15.07.15	24.07.15	03.08.15
D	fr & fv	lg	12.05.15	31.05.15	12.06.15	29.06.15	05.07.15	15.07.15	24.07.15	03.08.15
D	fr & fv	sg	12.05.15	01.06.15	12.06.15	29.06.15	05.07.15	15.07.15	24.07.15	03.08.15
D	fr & fv	cf	12.05.15	01.06.15	12.06.15	29.06.15	05.07.15	15.07.15	24.07.15	03.08.15
E	fr & fv	all	12.05.15	31.05.15	13.06.15	29.06.15	05.07.15	15.07.15	24.07.15	03.08.15
F	fr & fv	all	04.06.16	16.06.16	23.06.16	01.07.16	08.07.16	19.07.16	28.07.16	04.08.16
G	fr & fv	all	04.06.16	16.06.16	23.06.16	01.07.16	08.07.16	19.07.16	28.07.16	04.08.16
H	fr & fv	all	04.06.16	16.06.16	23.06.16	01.07.16	08.07.16	19.07.16	28.07.16	04.08.16

Abbreviations: blo., blocks A‐H; cf, closed forest; fr, flower resource; fv, flower visitors; hab, habitat; inspection passes 1–8; dates, dd.mm.yy; lg, large gap; param, parameter; sg, small gap.

Experimental approaches with potted, attractive food plants have proven to be effective in detecting the effects of and on flower visitors (Steffan‐Dewenter et al., 2002; Woodcock et al., 2014; Zurbuchen et al., 2010). Six native *Campanula* species occurred in the study area. To demonstrate their utility as phytometers, the list of plants below provides the light requirements, most important habitats, and habitat category in which the species occurred in the study area. The Ellenberg indicator value of light (L) represents the ecological characteristics of Central European plants in relation to the relative light availability in the field, ranging from one (occurring only in deep shadow) to nine (occurring only in full sunlight) (Ellenberget al., 1991). The list is arranged according to this indicator value. The most important habitats of the species are specified by BfN (1999). Finally, the occurrence in one of the habitat categories in the study area was described. The immediate surrounding area was defined as a square with a 30 m × 30 m side length centered on the pots containing *Campanula*.

- *C. patula*: L = 8; habitats are fresh meadows and pastures; flowered in the surrounding area in large and small gaps.
- *C. glomerata*: L = 7; habitats are dry and semi‐dry grasslands; flowered in the study area in large gaps, but not in the immediate surrounding area.
- C. *rotundifolia*: L = 7; habitats are rock sides, wall and scree vegetation, fresh meadows and pastures, dry and semi‐dry grasslands, deciduous and coniferous forests of acid and nutrient‐poor soils, heathlands, *Nardus* grasslands, and forests and shrubs of dry and warm locations; flowered in the surrounding area in large and small gaps.
- C. *rapunculoides*: L = 6; habitats are fields, field margins, ruderal vegetation, forests and shrubs of dry and warm locations; flowered in small gaps, but not in the immediate surrounding area, and in surrounding area in large gaps.
- *C. persicifolia*: L = 5; habitats are field margins, forests and shrubs of dry and warm locations; flowered in the surrounding area in large and small gaps and closed forest.
- *C. trachelium*: L = 4; habitats are deciduous and fir forests; flowered in the surrounding area in large and small gaps and closed forest.

We grew these six *Campanula* species from autochthonous seeds in pots in an open greenhouse under standardized field conditions: one plant per pot, same pot size, same soil substrate in the pots, and the same water and light conditions within a given species. We cultivated the plants from seeds for the year 2015 and new ones for 2016. The pots of one species were placed adjacent to each other beside the groups of pots of the other species. The pots of one species were placed in all three habitat categories of a block when the first flower opened. We returned the pots with one *Campanula* species to controlled garden conditions after the last flower had faded or at the latest when the seed pods opened. In the garden, the species received light according to their needs. As a result, the pots with the *Campanula* plants were only exposed to the influence of the different habitat categories during the flowering period.

Six pots of each species per site were considered sufficient to lure flower‐visiting insects (Sowig, 1989). All pots were placed adjacent to each other in the center of the study sites. We controlled predation, especially by slugs, with slug pellets.

### 2.3 Flower supply

Every site block was inspected eight times per year. During these eight inspection passes between May and the beginning of August in 2015 and 2016, we recorded the flower supply in the surrounding area (for specific dates, see Table 2). The surrounding area was defined as a square with a 30 m × 30 m side length centered around the pots with *Campanula*. To measure flower supply, we recorded the coverage of all indigenous flowers in cm² and the number of flowering plants. We summed up the flower cover in cm^2^ per 900 m^2^.

### 2.4 Flower visitors

During the eight inspections between May and August of 2015 and 2016, when conditions were dry and temperatures 20–28℃, we counted the individual visits of potential *Campanula* pollinators in the three habitat categories of the eight site blocks. At each inspection pass, we counted the flower‐visiting insects of all flowering *Campanula* plants for one minute, repeated this four times, and then summed up the values. Diptera, bees with body size greater than one cm (large bees) and bees with body size less than one cm (small bees) occurred in statistically evaluable numbers. Beetles, butterflies, and other flower‐visiting insect groups were included with flies, small and large bees in the category “all flower‐visiting insects.”

### 2.5 Pollination success

The pots with the *Campanula* species were returned to controlled conditions in a garden after the last flower had faded and before the first seed capsule had burst open. The timing of this return also minimized the influence of herbivores (snails). To test the pollination success in the different habitat categories, from the six pots in a plot, we collected ten fruits of each *Campanula* species and counted the number of seeds per fruit.

The six *Campanula* species in the study area have been deemed self‐incompatible (Gadella, 1964; Stephenson et al., 2000). However, depending on the presence of pollinators, self‐compatible and even spontaneous selfing plants of the genus *Campanula* may exist (Inoue & Amano, 1986; Stephenson et al., 2000). Therefore, we verified the self‐incompatibility of our *Campanula* species. For this purpose, we excluded flower visitors on one flower of each species with nylon stockings. Later, we determined if these capsules contained seeds.

### 2.6 Statistics

We performed two separate linear mixed‐effect models to estimate the habitat effect on the flower resources and the number of flowering species in the surrounding area. We added the eight site blocks as random effects in the models due to the experimental design.

Similarly, we analyzed the habitat effect on the occurrence of the four different flower visitor groups. The first group included all recorded flower visitors, which we then divided into the number of small bees, large bees, and flies. For each visitor group, we used a generalized linear mixed‐effect model (family=“poisson”) in which the number of visitors was used as response variables and the habitat categories as predictors. To control for the design, we again added the eight site blocks as random effects in the models.

To analyze the pollination success, we employed a generalized linear mixed model (family=“poisson”) for each of the six *Campanula* species. Here, we used the number of seeds as the response variable and the habitat categories as predictors. Similar to the models described earlier, we added the eight site blocks to control for the design.

We used the closed forest as the intercept because the deviation from the closed forest to the forest gaps is the effect of interest.

## 3 RESULTS

### 3.1 Flower supply

Flower cover in the area surrounding large gaps was significantly greater (estimate, 5,606) than in the closed forest. In contrast, flower cover in the area surrounding closed forest and small gaps did not differ significantly (Table 3). The number of flowering plant species in the surrounding area was significantly greater in the forest gaps than in the closed forest (estimated at 12.28 and 2.25, respectively; Table 3).

**TABLE 3:** The linear mixed‐effect models shows the effect of habitat category on the flower cover in the surrounding areas and on the number of flowering species in the surrounding areas. We set the closed forest as intercept (ic) throughout to show the deviation (estimate) to the large and the small gap

Response	Fixed effects			Random effects		
	Estimate	*SE*	*p*	Group	Variance	*SD*
Flower cover surroundings						
Closed forest (ic)	532	702	.46	Block	945,564	972
Small gap	−55	866	.95			
Large gap	5,606	866	<.001***			
Number flowering species surroundings						
Closed forest (ic)	1.16	0.84	.19	Block	3.24	1.80
Small gap	2.25	0.77	<.01**			
Large gap	12.28	0.77	<.001***			

Abbreviations: *p*, probability; *SD*, standard deviation; *SE*, standard error.

* *p* < .05

** *p* < .01

*** *p* < .001.

**FIGURE 2:**
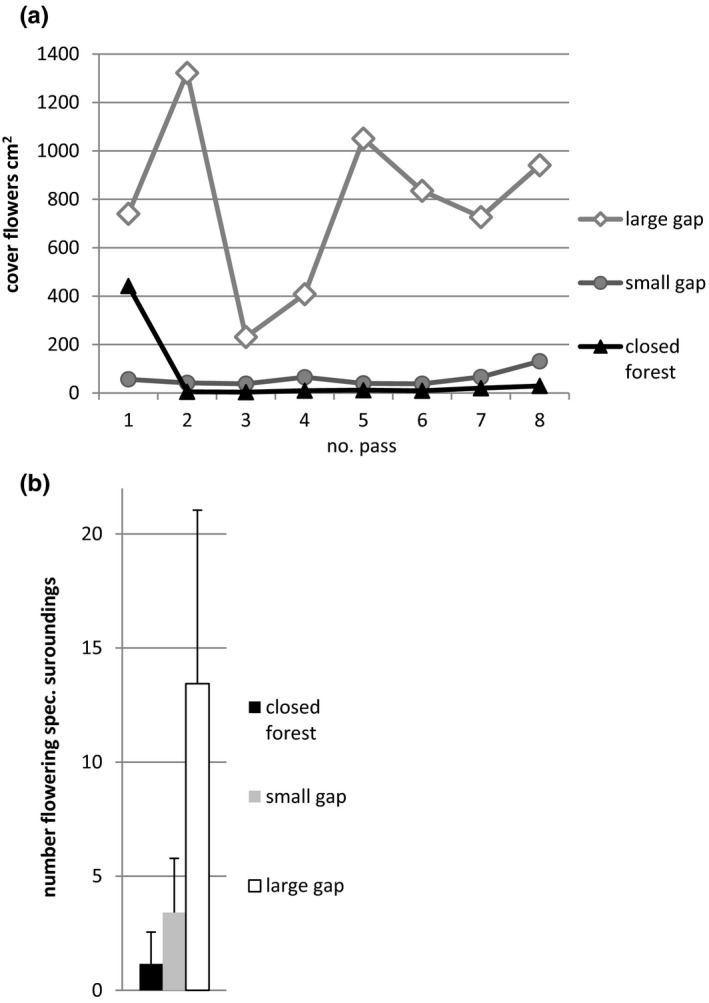
(a) Mean flower cover (cm^2^) of the surrounding areas (30 m × 30 m) of the three habitat categories throughout the eight inspection passes. The dates of inspection passes are in Table 2. (b) The number of flowering plant species (mean and standard deviations) in the surrounding areas (30 m × 30 m) of the two forest gaps was significantly different from that of closed forest

When we compare the absolute numbers, we see that the mean flower cover in the large gap was 6,138 cm^2^ in 900 m^2^ surrounding area, while it was lower in small gaps (477 cm^2^) than in closed forest (532 cm^2^). Especially in the first inspection pass, the flower cover in the forest was very high and changed the ranking of small gaps and closed forest (Figure 2a). The mean number of flowering species of the surrounding area (30 m × 30 m) was 13.4 in large gaps, 3.4 in small gaps, and 1.2 in the closed forest (Figure 2b).

### 3.2 Flower visitors

**FIGURE 3:**
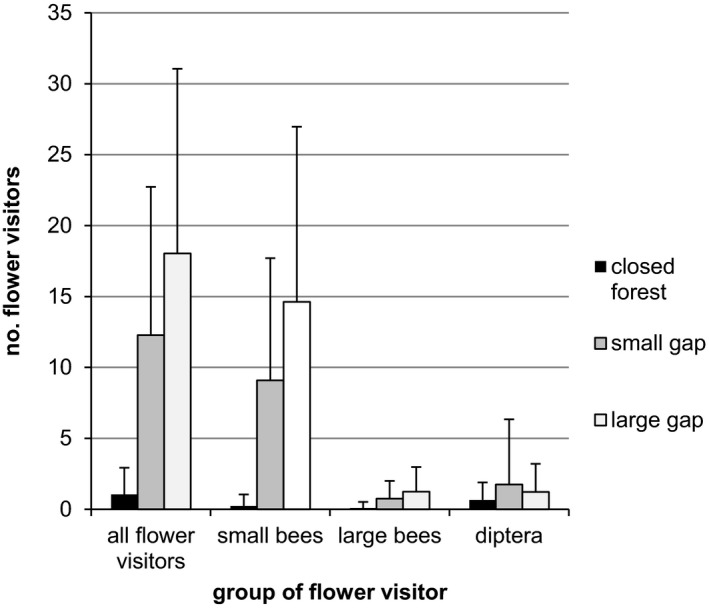
The number of flower visitors (mean and standard deviation) in different groups in the three habitat categories. All flower visitors also included butterflies, beetles, and other insect groups, which were only recorded in small numbers. In all groups of flower visitors, the numbers of small and large gaps differed significantly from those of closed forest

The numbers of all flower visitor groups were significantly more frequent in two‐gap habitat categories than in closed forest (Table 4). The deviation between the forest gaps and the closed forest was greatest for the small bees (estimated at 4.1 and 3.7, respectively) and smallest for the flies (estimated at −0.62 and 0.98, respectively). The large gaps were a greater distance from the closed forest than the small gaps at all flower visitors and small and large bees. Only the model for the number of flies showed a greater distance to small (estimate 0.98) than to large gaps (estimate −0.62; Table 4). All groups of flower visitors showed the lowest abundances in the closed forest (Figure 3). All flower visitors and small and large bees were most frequent in large gaps, while flies were more frequent in small gaps (Figure 3). In the closed forest, the absolute abundances of all visitor groups were less than one individual per count (Figure 3). Flies were in the closed forest most frequent, while bees dominated the forest gaps.

**TABLE 4:** The generalized linear mixed‐effect models shows the effect of habitat category on the number of flower visitors in different groups

Response flower visitors	Fixed effects			Random effects		
	Estimate	*SE*	*p*	Group	Variance	*SD*
No. all visitors						
Closed forest (ic)	0.01	0.15	.089	Block	0.06	0.25
Small gap	2.46	0.13	<.001***			
Large gap	2.85	0.13	<.001***			
No. small bees						
Closed forest (ic)	−1.48	0.27	<.001***	Block	0.06	0.25
Small gap	3.66	0.26	<.001***			
Large gap	4.13	0.26	<.001***			
No. large bees						
Closed forest (ic)	−2.45	0.43	<.001***	Block	0.17	0.41
Small gap	2.08	0.43	<.001***			
Large gap	2.58	0.42	<.001***			
No. flies						
Closed forest (ic)	−0.64	0.28	<.05*	Block	0.44	0.67
Small gap	0.98	0.18	<.001***			
Large gap	0.62	0.19	<.001**			

Abbreviations: *p*, probability; *SD*, standard deviation; *SE*, standard error.

* *p* < .05

** *p* < .01

*** *p* < .001.

### 3.3 Pollination success

**FIGURE 4:**
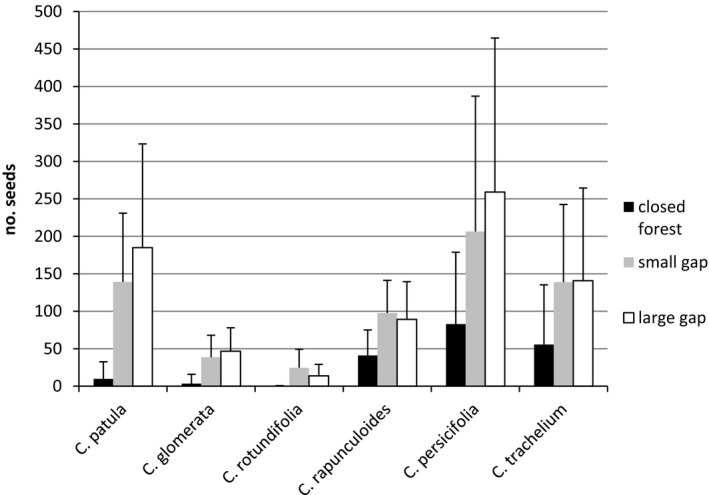
The number of seeds produced in 10 capsules (mean and standard deviation) of the six *Campanula* species in the three habitat categories. We arranged the *Campanula* species in decreasing order of their Ellenberg indicator value of light (Ellenberg et al., 1991). In all species, the number of seeds in small and large gaps differed significantly from that of closed forest

The capsules excluded from pollination did not produce any seeds, confirming that spontaneous selfing did not occur. Forest gaps and their size positively influenced the number of seeds in all six studied *Campanula* species (Table 5). All *Campanula* species had the lowest number of seeds in the closed forest. Most species had the highest number of seeds in large gaps, whereas *C. rotundifolia* and *C. rapunculoides* produced more seeds in the small gaps (Figure 4). The deviation of the seed set in forest gaps from that in the closed forest was larger for the light‐demanding species *C. patula, C. glomerata,* and *C. rotundifolia* (estimated from 2.41 to 5.77) than for the shade‐tolerant species *C. rapunculoides, C. persicifolia,* and *C. trachelium* (estimated from 0.78 to 1.14; Table 5). Species with an Ellenberg indicator value of light of 7 or 8—*C. rotundifolia, C. glomerata,* and *C. patula*—produced a mean of fewer than 10 seeds in the closed forest, while *C. rapunculoides* (L = 6), *C. persicifolia* (L = 5), and *C. trachelium* (L = 4) produced a mean of 41 and 83 seeds, respectively. The highest number of mean seeds was 259 for *C. persicifolia* in large gaps (Figure 4).

**TABLE 5:** The generalized linear mixed‐effect models shows the effect of habitat category on the number of seeds of the six *Campanula* species. We arranged the species in decreasing order of Ellenberg indicator value of light (Ellenberg et al., 1991)

Seed response	Fixed effects			Random effects		
	Estimate	*SE*	*p*	Group	Variance	*SD*
*C. patula*						
Closed forest (ic)	2.20	0.16	<.001***	Block	0.18	0.43
Small gap	2.69	0.04	<.001***			
Large gap	3.00	0.04	<.001***			
*C. glomerata*						
Closed forest (ic)	1.15	0.17	<.001***	Block	0.19	0.44
Small gap	2.41	0.06	<.001***			
Large gap	2.65	0.06	<.001***			
*C. rotundifolia*						
Closed forest (ic)	−2.67	0.43	<.001***	Block	0.16	0.40
Small gap	5.77	0.41	<.001***			
Large gap	5.24	0.41	<.001***			
*C. rapunculoides*						
Closed forest (ic)	3.69	0.1	<.001***	Block	0.05	0.23
Small gap	0.87	0	<.001***			
Large gap	0.78	0	<.001***			
*C. persicifolia*						
Closed forest (ic)	4.34	0.13	<.001***	Block	0.14	0.37
Small gap	0.91	0.15	<.001***			
Large gap	1.14	0.01	<.001***			
*C. trachelium*						
Closed forest (ic)	3.56	0.44	<.001***	Block	1.53	1.24
Small gap	0.80	0.02	<.001***			
Large gap	0.89	0.01	<.001***			

Abbreviations: *p*, probability; *SD*, standard deviation; *SE*, standard error.

* *p* < .05

** *p* < .01

*** *p* < .001.

## 4 DISCUSSION

### 4.1 Flower supply

It is well known that light positively affects flower formation even in forest plants (Cao et al., 2017; Cunningham, 1997; Killkenny & Galloway, 2008). This observation is consistent with expectations that the number of flowering species would be higher in small gaps (glades) than in closed forest and highest in large gaps (clearings). However, flower cover was only significantly higher in the large gaps than in the closed forest, but not in the small gaps. We attribute this to the high flower cover of spring geophytes in the “closed deciduous forest” in the first inspection pass in May, in addition to a large standard deviation in all inspection passes (Figure 2a). Thus, when forest gaps are overgrown, flower supply declines.

### 4.2 Flower visitors

If forest gaps become overgrown into closed forests, all the groups of flower visitors studied showed declines in their flower visits. These declines were particularly clear for small bees while hardly noticeable for flies (Table 4). Temperature also varied in each habitat category (shading). We assume that temperature explains the differences in abundances between small bees, large bees, and flies. In warm temperatures, bees are dominant flower visitors, while flies are abundant at more moderate temperatures (Adedoja et al., 2018; Corbet et al., 1993; Herrera, 1997; Hodkinson, 2005; Ssymank, Keams, et al., 2011). However, large bees are not a uniform group with regard to their temperature requirements. Bumblebees may also fly at very cool temperatures. In contrast, certain large bees such as *Megachile* spec. or *Melitta haemorrhoidalis* require warmer temperatures (Westrich, 2018).

Similarly, flower visitors were found to more frequently visit *Campanulastrum americanum* (Killkenny & Galloway, 2008) and *Hosta ventricosa* (Cao et al., 2017) in sunny and open patches than in shaded and forested ones. In contrast, Hansen and Totland (2006) found no difference in the number of flower visitors of *Campanula persicifolia* between forest and meadow habitats in Norway. However, they had counted mainly hoverflies, muscoid flies, and few bumblebees. We interpret this as an indication that temperatures in Norway were similarly cool in both forest and meadow, in contrast to the habitats we studied (Adedoja et al., 2018; Ssymank, Kearns, et al., 2011).

### 4.3 Pollination success

The results clearly showed that the overgrowth of forest gaps into closed forests negatively affected the seed production of all studied *Campanula* species. As expected, the negative influence was stronger in light‐demanding species, such as *C*. *patula*, *C*. *glomerata,* and *C*. *rotundifolia*, than in less light‐demanding species, such as *C*. *persicifolia*, *C*. *trachelium,* and *C*. *rapunculoides*. Although *C. patula* has a higher Ellenberg indicator value for light than *C. glomerata* and *C. rotundifolia*, it demonstrates greater plasticity with respect to light and a generally higher seed number. If forest gaps are overgrown into closed forests, the extremely low seed production becomes a limiting factor for reproduction (extinction debt). Moreover, spontaneous selfing resulting in any seed set could not be detected when excluding pollinators, supporting the validity of our results.

Goodell et al. (2010) revealed similar effects for *Lonicera maacki* in edge and interior forest habitats, Killkenny and Galloway (2008) for *Campanulastrum americanum* in the sun and the shade and Cao et al. (2017) for *Hostea ventricosa* in open and closed forest habitats. In contrast, no significant difference was detected in the number of *Campanula persicifolia* seeds between forest and meadow habitats in Norway (Hansen & Totland, 2006). In the Norwegian study, the number of seeds per fruit was strongly pollen‐limited in both habitat categories, possibly indicating that flower visitors were crucial for pollination. In contrast, regarding groups of flower visitors, our study showed differences in pollination success, whereas the Norwegian study did not.

### 4.4 Relevance to conservation

In a previous study, we showed that from 1945 to 2015, 81% of forest gaps larger than 150 m^2^ became closed forest areas (Braun‐Reichert & Poschlod, 2018). If we place the results of the habitat categories in this temporal context, then the overgrowth of forest gaps has clear negative effects on the flower supply, the number of flower visitors of *Campanula* species, and the number of seeds they produce.

It is reasonable to suppose that the effects regarding *Campanula* flower supply and the number of seeds are transferable to other plant species of light and open canopy forests (Barbier et al., 2008; Hurskainena et al., 2017). They are often listed as fringe species, for example, typical species of the class Trifolio‐Geranietea. Therefore, we can assume that typical plants of open forests are not only declining due to habitat loss but also because of reduced or missing seed sets, which eventually may result in the extinction of local populations. The historical age of forest gaps plays an especially important role in plant species richness (Husakova & Münzbergova, 2014). For pollinators, forest gaps can play an important role as a small natural feature in closed forest, from which flowers in the surrounding forest are also visited (Poschlod & Braun‐Reichert, 2017). In addition, an opening of the tree canopy positively affects the number of other arthropod species (Bussler, 2016; Müller et al., 2007). It is known that butterflies, in particular, have a high diversity in open and coppice forests (Fartmann et al., 2013). Furthermore, light forests and forest gaps are important habitats for other animal groups such as birds or bats (Dietz et al., 2016; Gatter, 2004). Hilmers et al. (2018) illustrated the importance of light in the initial stage of forest succession for the diversity of various organism groups. Forest gaps increase habitat diversity, structural complexity, and faunal and floral species diversity (Muscolo et al., 2014). Historically open forests should be preserved as a part of the European cultural landscape and as important habitat for flora and fauna, especially pollinators.

Only a few decades earlier, open forests and forest gaps were much more common in the cultural landscape of Central Europe (Poschlod, 2017). Now, historical forms of forest use have been abandoned, and nitrogen deposition has increased (Hampicke, 2018; Poschlod, 2017; Stuber & Bürgi, 2011; Verheyen et al., 2012). Even naturally formed gaps are growing over faster than they would without the heavy nitrogen inputs. Especially on marginal sites like rocky heads, where only certain tree species could grow very slowly, are now colonized by atypical tree species with very dense canopies like *Fagus sylvatica*. Therefore, the political demand for a reduction of nitrogen inputs must be continuously asserted (Sutton et al., 2011).

Maintenance is necessary to preserve forest gaps of high ecological value because, without human intervention, they will close (Braun‐Reichert & Poschlod, 2018; Bussler, 2016). However, simple thinning through logging often leads to undesirable effects such as strong growth of *Rubus* spp. or other nutrient‐indicating plants. A significant reduction of N deposition is necessary for the long term to preserve forest gaps (Sutton et al., 2011; Verheyen et al., 2012). Historical forms of land use in forests such as forest grazing or coppice are complex (in terms of target species, type of grazing animal, and intensity and duration of grazing) and labor‐intensive and therefore not easy to implement (Bärnthol, 2003; Liegl & Dolek, 2008; Poschlod, 2017; Rackham, 2003; Rupp & Michiels, 2020; Zahn et al., 2014). However, grazing animals and coppicing would remove nutrients which contributes to the openness of the forest (Bärnthol, 2003; Berendse, 1985; Marrs et al., 2020). The new development of pristine forests is often set as a conservation goal, which is easier to manage but seemingly contradicts forest gap management. Only seemingly, because forest gaps are small natural features that do not occupy large areas in contrast to pristine forests. However, the implementation of both concepts would greatly increase the diversity of an area and its ecological and nature conservation value.

## CONFLICT OF INTEREST

None declared.

## AUTHOR CONTRIBUTIONS

**Ralf Braun‐Reichert:** Conceptualization (equal); Data curation (lead); Formal analysis (equal); Investigation (lead); Methodology (equal); Project administration (equal); Resources (lead); Supervision (equal); Validation (equal); Visualization (lead); Writing – original draft (lead); Writing – review and editing (lead). **Peter Poschlod:** Conceptualization (equal); Methodology (equal); Project administration (equal); Resources (equal); Supervision (supporting); Visualization (supporting); Writing – review and editing (supporting). **Sven Rubanschi:** Formal analysis (equal); Methodology (equal); Software (supporting); Validation (equal); Writing – review and editing (supporting).

## Data Availability

The data of flower supply, flower visitors, and number of seeds that support the findings of this study are openly available in Dryad at http://doi.org/10.1002/ece3.7965.
